# Therapeutic strategy with artificially-designed i-lncRNA targeting multiple oncogenic microRNAs exhibits effective antitumor activity in diffuse large B-cell lymphoma

**DOI:** 10.18632/oncotarget.9237

**Published:** 2016-05-09

**Authors:** Yinghan Su, Bin Sun, Xuejing Lin, Xinying Zhao, Weidan Ji, Miaoxia He, Haihua Qian, Xianmin Song, Jianmin Yang, Jianmin Wang, Jie Chen

**Affiliations:** ^1^ School of Life Science, University of Liverpool, Liverpool, L3 4PH, UK; ^2^ Department of Molecular Oncology, Eastern Hepatobiliary Surgical Hospital & National Center of Liver Cancer, Second Military Medical University, Shanghai 200438, China; ^3^ Department of Hematology & Pathology, Changhai Hospital, Second Military Medical University, Shanghai 200168, China

**Keywords:** diffuse large B-cell lymphoma, artificial long non-coding RNA, oncogenic microRNA, xenograft model, therapeutic strategy

## Abstract

In diffuse large B-cell lymphoma (DLBCL), many oncogenic microRNAs (OncomiRs) are highly expressed to promote disease development and progression by inhibiting the expression and function of certain tumor suppressor genes, and these OncomiRs comprise a promising new class of molecular targets for the treatment of DLBCL. However, most current therapeutic studies have focused on a single miRNA, with limited treatment outcomes. In this study, we generated tandem sequences of 10 copies of the complementary binding sequences to 13 OncomiRs and synthesized an interfering long non-coding RNA (i-lncRNA). The highly-expressed i-lncRNA in DLBCL cells would compete with the corresponding mRNAs of OncomiR target genes for binding OncomiRs, thereby effectively consuming a large amount of OncomiRs and protecting many tumor suppressor genes. The *in vitro* experiments confirmed that the i-lncRNA expression significantly inhibited cell proliferation, induced cell cycle arrest and apoptosis in DLBCL cell lines, mainly through upregulating the expression of PTEN, p27^kip1^, TIMP3, RECK and downregulating the expression of p38/MAPK, survivin, CDK4, c-myc. In the established SUDHL-4 xenografts in nude mice, the treatment strategy involving adenovirus-mediated i-lncRNA expression significantly inhibited the growth of DLBCL xenografts. Therefore, this treatment would specifically target the carcinogenic effects of many OncomiRs that are usually expressed in DLBCL and not in normal cells, such a strategy could improve anti-tumor efficacy and safety and may be a good prospect for clinical applications.

## INTRODUCTION

Diffuse large B-cell lymphoma (DLBCL) is one of the most common types of non-Hodgkin's lymphoma (NHL) with poor prognosis. During recent years, optimized stratified treatment regimens based on clinical stage and international prognostic index (IPI) together with post-chemotherapy hematopoietic stem cell transplantation or molecular targeted therapy have been gradually adopted in clinical practice and have provided a focus and direction for advances in DLBCL treatment [1–3]. These comprehensive treatment regimens prolong the remission and disease-free survival of patients with DLBCL, thereby improving the overall treatment outcomes. However, relapse and drug resistance remain the leading causes of death in patients with DLBCL; hence, we must find new treatment strategies to address the shortcomings of the few currently available alternative treatment options and poor outcomes of DLBCL treatment and should strive to achieve complete remission or good long-term survival in patients.

Micro-ribonucleic acids (microRNAs; miRNAs) regulate a large number of human genes. miRNAs are small molecules that are present in large numbers, have characteristics of wide distribution and significant effect, and are an integral part of the gene regulatory network [4, 5]. In 2002, one research group conducted a study of B-cell chronic lymphocytic leukemia and observed a loss of expression of miR-15 and miR-16, thus providing the first evidence of an association between miRNAs and tumors [6]. Subsequently, an increasing number of studies have observed abnormal miRNA expression in many tumors, and this appears to associate closely with the regulation of tumor development and progression [7, 8]. Therefore, miRNAs have become an important target of targeted cancer therapies. Regarding DLBCL development and progression, many miRNAs are found to directly affect DLBCL differentiation, malignant transformation, and the regulation of sensitivity to chemical drugs, thus affecting the treatment outcomes and prognoses of patients. In particular, miRNAs highly expressed in DLBCL can directly or indirectly inhibit the expression and function of certain tumor suppressor genes, and thus, these miRNAs are also known as oncogenic miRNAs (OncomiRs). Studies have reported that many miRNAs are highly expressed in DLBCL, such as miR-17-5p, miR-18a-5p, miR-19b-3p, miR-20a-5p, miR-21, miR-23a, miR-27a, miR-28-5p, miR-106a-5p, miR-125a/5b, miR-146a, miR-148a, miR-150, miR-155, miR-181a-5p, miR-200c, miR-212, miR-214-5p, miR-221/222, miR-324-5p, miR-339-3p, miR-363, miR-487b, miR-513, miR-518a, miR-770-5p, miR-5586-5p, miR-10393-3p, NOVELM00203M, etc. [9–11]. Based on the mechanism of miRNA function, the OncomiRs can target a varity of onco-suppressor genes and inhibit their post-transcriptional expression. For example, PTEN (phosphate and tensin homolog) is a tumor suppressor gene, which was regulated by several miRNAs, such as miR-21 [12], miR-155 [13], miR-221/222 [14], and miR-17~92 cluster [15]. In DLBCL, the high-expressed OncomiRs bind to PTEN mRNA and inhibit its expression. Loss of PTEN leads to activation of the PI3K/AKT/mTOR pathway, then promotes the proliferation and progression of DLBCL, and shortens the progression-free survival (PFS) and overall survival (OS) in patients with DLBCL. These miRNAs comprise a promising new class of molecular targets for the treatment of DLBCL. miR-21 is an OncomiR that affects the development and progression of DLBCL, and its expression correlates positively with the tumor cell proliferation index. miR-21 inhibitor interferes with miR-21 expression, thereby inhibiting the proliferation and invasion of DLBCL cells and inducing apoptosis [16]. Therefore, miR-21 regulates the malignant biological behaviors of DLBCL, and accordingly, anti-miR-21 approach may become a useful DLBCL treatment strategy. Studies have confirmed that the use of miRNA inhibitors or antisense sequences to block OncomiR expression or inhibit OncomiR function can inhibit the growth of many tumors [17–20].

However, most current therapeutic studies have focused on a single miRNA, with limited treatment outcomes. These unsatisfactory treatment outcomes might occur because miRNAs target many genes in the setting of complex regulatory mechanisms. One miRNA can target many genes, and one target gene might be regulated by many miRNAs. Furthermore, many miRNA molecules are involved in tumor development and progression through the extensive regulation of target gene expression or through effects on many signaling pathways. Therefore, cancer cells can easily regain proliferative activity through alternative pathways. As a result, interventions that target many miRNAs with different or complementary mechanisms and inhibit multiple signaling pathways will be more effective for cancer treatment.

Intracellular miRNAs bind to the messenger RNAs (mRNAs) of target genes with complementary sequences to induce mRNA degradation or inhibit mRNA translation, thereby exerting their role as post-transcriptional regulators of target genes [21]. Based on the mechanisms of miRNA functions, we selected those that are highly expressed in DLBCL, including miR-21, miR-155, miR-221/222, miR-125a-5p/125b, and miR-146a/146b-5p, as well as the miR-17-92 family members miR-17, miR-19a/19b, and miR-20a/20b; subsequently, we generated tandem sequences of 10 copies of the antisense sequences to these miRNA seed sequences and synthesized an interfering long non-coding RNA (i-lncRNA). This i-lncRNA contained multiple repeat sequences capable of binding to the OncomiRs aforementioned; accordingly, the i-lncRNA would compete with the corresponding mRNAs of target genes for OncomiR binding, thereby effectively consuming a large amount of OncomiRs in the cells. This process would interfere with many OncomiRs by protecting the targeted tumor suppressor genes and promoting anti-tumor effects. This treatment strategy would specifically target the carcinogenic effects of many OncomiRs that are usually not expressed in normal cells. Therefore, such a treatment strategy could improve anti-tumor efficacy and safety and is a good prospect for clinical applications.

## RESULTS

### Efficiency of adenovirus infection and interaction between i-lncRNA and OncomiRs

**Figure 1:**
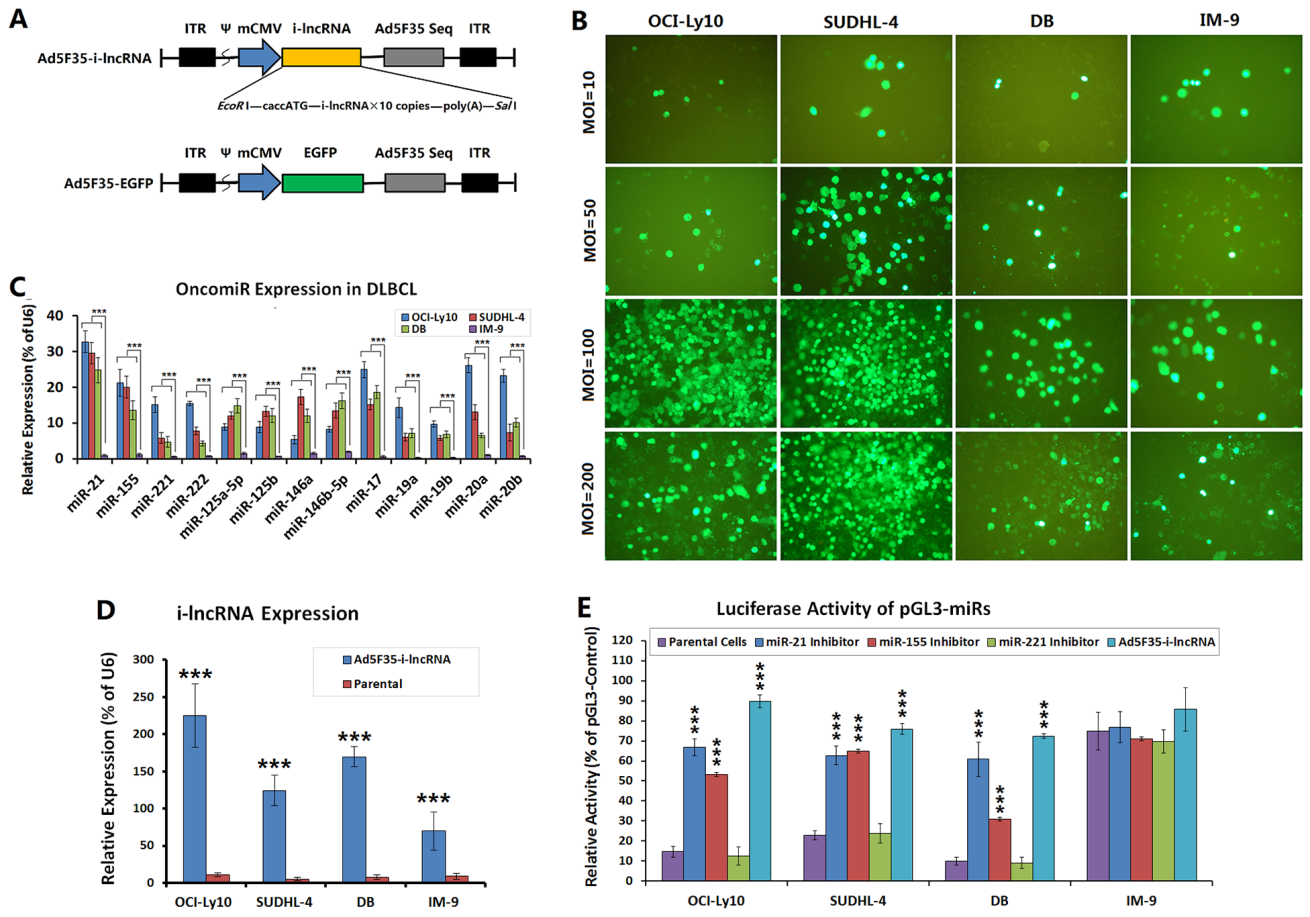
Efficiency of adenovirus infection and expression of OncomiRs and i-lncRNA **A.** Schematic diagrams of the adenoviruses, the expression cassette of whole-length encoding sequence of i-lncRNA or the EGFP gene was inserted into the *Eco*RI + *Sal*I sites of adenovirus E1 region, to generate the recombinant adenovirus Ad5F35-i-lncRNA or Ad5F35-EGFP. ITR: inverted terminal repeats; ψ: adenovirus 5 packaging signal; mCMV: mouse cytomegalovirus promoter. **B.** DLBCL cell lines (OCI-Ly10, SUDHL-4, DB) and normal human peripheral B cell line (IM-9) were cultured in 96-well plates at a density of 1 × 10^4^ cells/100 μL/well for 24 h, then infected with Ad5F35-EGFP at MOIs of 10, 50, 100 and 200 pfu/cell. Another 48 h later, the percentages of EGFP-positive cells were observed and counted under a fluorescent microscope; original magnification: 200×. **C.** The aforementioned cell lines were cultured in 6-well plates at a density of 1 × 10^5^ cells/100 μL/well for 48 h, then harvested for isolation of total RNA, which was used to detect the expression levels of the indicated OncomiRs by qRT-PCR; ***P<0.001. **D.** Cell lines were cultured in 6-well plates at a density of 1 × 10^5^ cells/100 μL/well for 24 h, then infected with Ad5F35-i-lncRNA at an MOI of 100 pfu/cell. Another 48 h later, cells were harvested for isolation of total RNA, which was used to detect the expression levels of i-lncRNA by qRT-PCR; ***P<0.001 compared with the corresponding parental cells. **E.** Cell lines were seeded into 24-well plates at a density of 5×10^5^ cells/well and transfected with miRNA inhibitors at a concentration of 100 nmol/L or infected with Ad5F35-i-lncRNA at MOIs of 10 to 200 pfu/cell. After incubation for 24 h, cells were co-transfected with 200 ng/well of pGL3-miRs together with 20 ng/well of pRL-TK using Lipofectamine 2000. At 48 h after transfection, cells were harvested and used to detect the relative luciferase activity with the Dual-Luciferase Reporter Assay, normalized with the activity of pGL3-Control in every cell line; ***P<0.001 compared with the corresponding parental cells.

Adenoviruses Ad5F35-i-lncRNA and Ad5F35-EGFP were constructed to express an interfering long non-coding RNA (i-lncRNA) and EGFP, respectively (Figure 1A). All cell lines were infected with Ad5F35-EGFP at MOIs of 10, 50, 100 and 200 pfu/cell to count the viral infection efficiency. At an MOI of 100 pfu/cell, the infection efficiencies were 86.33%, 91.51%, 82.56%, and 78.52% in the OCI-Ly10, SUDHL-4, DB, and IM-9 cell lines, respectively (Figure 1B). MOIs of 200 pfu/cell induced more cell death. Therefore, a standard MOI of 100 pfu/cell was used in the cytological experiments.

Quantitative RT-PCR (qRT-PCR) was performed to detect OncomiR expression in the experimental cells. The expression levels of miR-21, miR-155, miR-221/222, miR-125a-5p/125b, miR-146a/146b-5p, miR-17, miR-19a/19b, and miR-20a/20b were significantly higher in the OCI-Ly10, SUDHL-4, and DB cells than in the IM-9 cells. Of these OncomiRs, the expression levels of miR-21, miR-155, miR-221/222, miR-17, miR-19a/19b, and miR-20a/20b were higher in OCI-Ly10 cells, whereas the expression levels of miR-21, miR-155, miR-125a-5p/125b, miR-146a/146b-5p, and miR-17 were higher in SUDHL-4 and DB cells (Figure 1C). The cells were infected with Ad5F35-i-lncRNA at an MOI of 100 pfu/cell and subjected to qRT-PCR to detect i-lncRNA expression. In accordance with the Ad5F35-EGFP-mediated EGFP expression, i-lncRNA expression was slightly higher in the OCI-Ly10, SUDHL-4, and DB cells than in the IM-9 cells, although this difference was not statistically significant. The uninfected cells exhibited no i-lncRNA expression (Figure 1D).

We confirmed the interactions between the OncomiRs and the complementary binding sequences within i-lncRNA by a luciferase assay. The luciferase activities of pGL3-miRs containing miR-21 and miR-155 binding sites were lower in OCI-Ly10, SUDHL-4 and DB cells than in IM-9 cells, and the inhibitors of miR-21 and miR-155 could increase the luciferase levels in DLBCL cells, but the control miR-221 inhibitor did not. The luciferase activities of pGL3-miRs in the Ad5F35-i-lncRNA-infected DLBCL cells were markedly increased, but only an unconspicuous upregulation IM-9 cells (Figure 1E), suggesting that the lncRNAi expression could protect the target gene from being inhibited by OncomiRs in DLBCL cells.

### Effect of i-lncRNA expression on DLBCL cell proliferation

**Figure 2:**
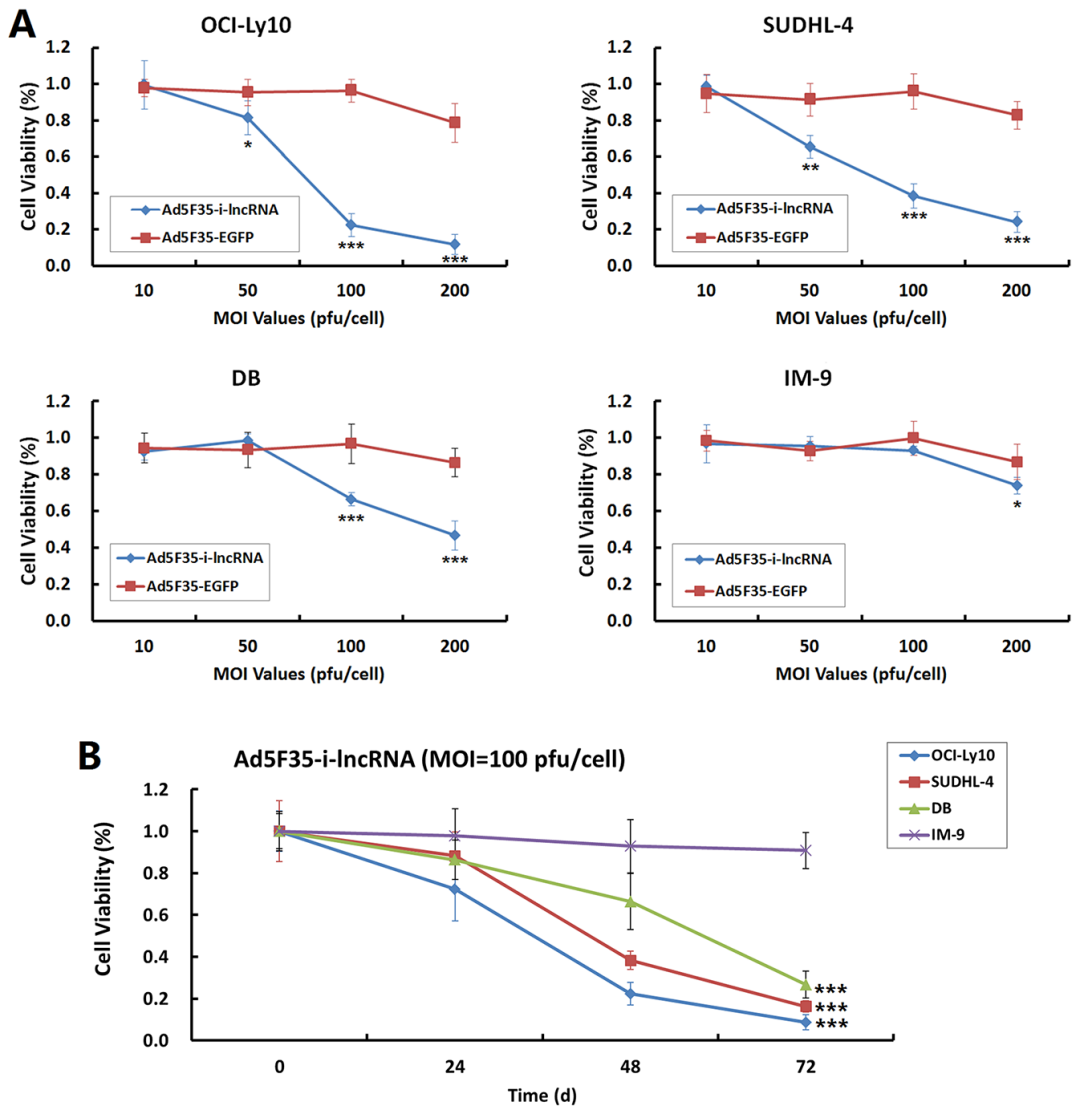
Inhibitory effect of i-lncRNA on cell proliferation **A.** The four cell lines were planted in 96-well plates at a density of 1 × 10^4^ cells/100 μL/well. After cultured for 24 h, the viruses Ad5F35-i-lncRNA and Ad5F35-EGFP were added at MOIs of 10 to 200 pfu/cell in a volume of 100 μL/well. Cells were continuously cultured for 48 h followed by CCK-8 assay analysis. A microplate reader was used to measure the optical density (OD) of each well at 490 nm, the data were used to plot cell viability curves; *P<0.05, **P<0.01 and ***P<0.001 compared with the Ad5F35-EGFP-infected group at the same MOI value. **B.** Cells were infected with Ad5F35-i-lncRNA at an MOI of 100 pfu/cell and cultured for 24 h, 48 h, and 72 h. The cell viability was measured as aforementioned; ***P<0.001 compared with IM-9 cells.

Cells were infected with Ad5F35-i-lncRNA and Ad5F35-EGFP at MOIs of 10, 50, 100 and 200 pfu/cell and subjected to CCK-8 assay to analyze the effect of i-lncRNA expression on cell proliferation. The results showed that Ad5F35-i-lncRNA infection significantly inhibited DLBCL cell proliferation, with a significant decrease in viability observed in OCI-Ly10 and SUDHL-4 cells. At an MOI of 100 pfu/cell, the viabilities of OCI-Ly10 and SUDHL-4 cells were reduced to less than 50%, and the viability of DB cells was reduced to less than 70%. The control adenovirus Ad5F35-EGFP had no significant inhibitory effect on proliferation in any cell line when MOIs were equal to and below 100 pfu/cell, but the viability of IM-9 cells was decreased significantly at an MOI of 200 pfu/cell (Figure 2A). At an MOI of 100 pfu/cell, Ad5F35-i-lncRNA had no significant effect on the viability of IM-9 cells, which remained more than 90% at every timepoint. In contrast, the Ad5F35-i-lncRNA-infected cancer cell lines showed gradually decreased viabilities with the prolonging of culture time (Figure 2B).

### Effect of i-lncRNA expression on DLBCL cell cycle and apoptosis

**Figure 3:**
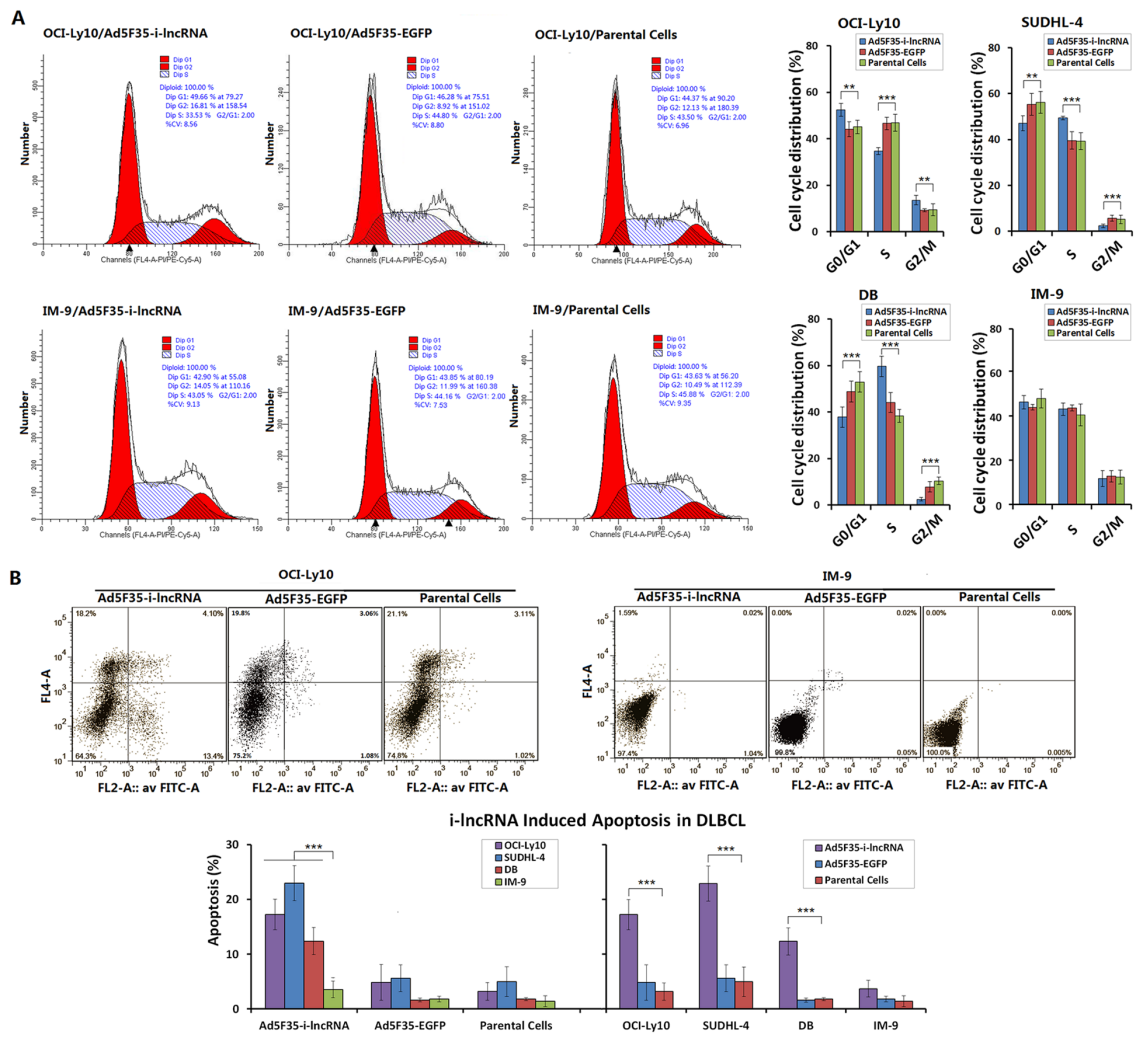
Inhibitory effect of i-lncRNA on cell cycle and apoptosis **A.** The four cell lines were cultured in 6-well plates at a density of 1 × 10^5^ cells/100 μL/well for 24 h, then infected with Ad5F35-i-lncRNA or Ad5F35-EGFP at an MOI of 100 pfu/cell. After continuously cultured for 48 h, cells were harvested and fixed in pre-chilled 75% ethanol, placed in a 4°C refrigerator overnight, washed in PBS twice, added the RNase-containing PI staining mixture and incubated in dark for 30 min. Cells were subjected to cell cycle analysis by flow cytometry; **P<0.01 and ***P<0.001. **B.** Cells were cultured and infected with the viruses as aforementioned, harvested 48 h later and stained with Annexin V/PI. Cell apoptosis was analyzed by flow cytometric analysis; ***P<0.001.

The IM-9 cells infected with Ad5F35-i-lncRNA and Ad5F35-EGFP at an MOI of 100 pfu/cell exhibited no significant changes in the frequencies of cell cycle phases or apoptotic cells. After DLBCL cells were infected with Ad5F35-i-lncRNA, however, higher frequencies of OCI-Ly10 cells were observed in the G0/G1 and G2/M phases, along with a significantly lower frequency of cells in the S phase; in contrast, SUDHL-4 and DB cells exhibited a slightly lower frequency of cells in the G0/G1 phase, a significantly lower frequency of cells in the G2/M phase, and a higher frequency of cells in the S phase. After infection with Ad5F35-EGFP, only OCI-Ly10 cells exhibited a higher frequency of cells in the G2/M phase (Figure 3A). After infection with Ad5F35-i-lncRNA, the DLBCL cells exhibited significant increases in apoptotic frequency, whereas after infection with Ad5F35-EGFP, no change in apoptosis was exhibited in every cell line (Figure 3B).

### Effect of i-lncRNA expression on target gene expression in DLBCL cells

We selected the representative target genes of OncomiRs for an additional Western blot analysis, including the tumor suppressors PTEN, p27kip1, metallopeptidase inhibitor 3 (TIMP3) and reversion-inducing-cysteine-rich protein with Kazal motifs (RECK) that are directly targeted by i-lncRNA, and the oncogenic factors p38/mitogen-activated protein kinases (MAPK), Survivin, cyclin-dependent kinase 4 (CDK4) and c-myc that are indirectly related with the changes of cancer cell malignant behaviors mediated by i-lncRNA expression.

**Figure 4:**
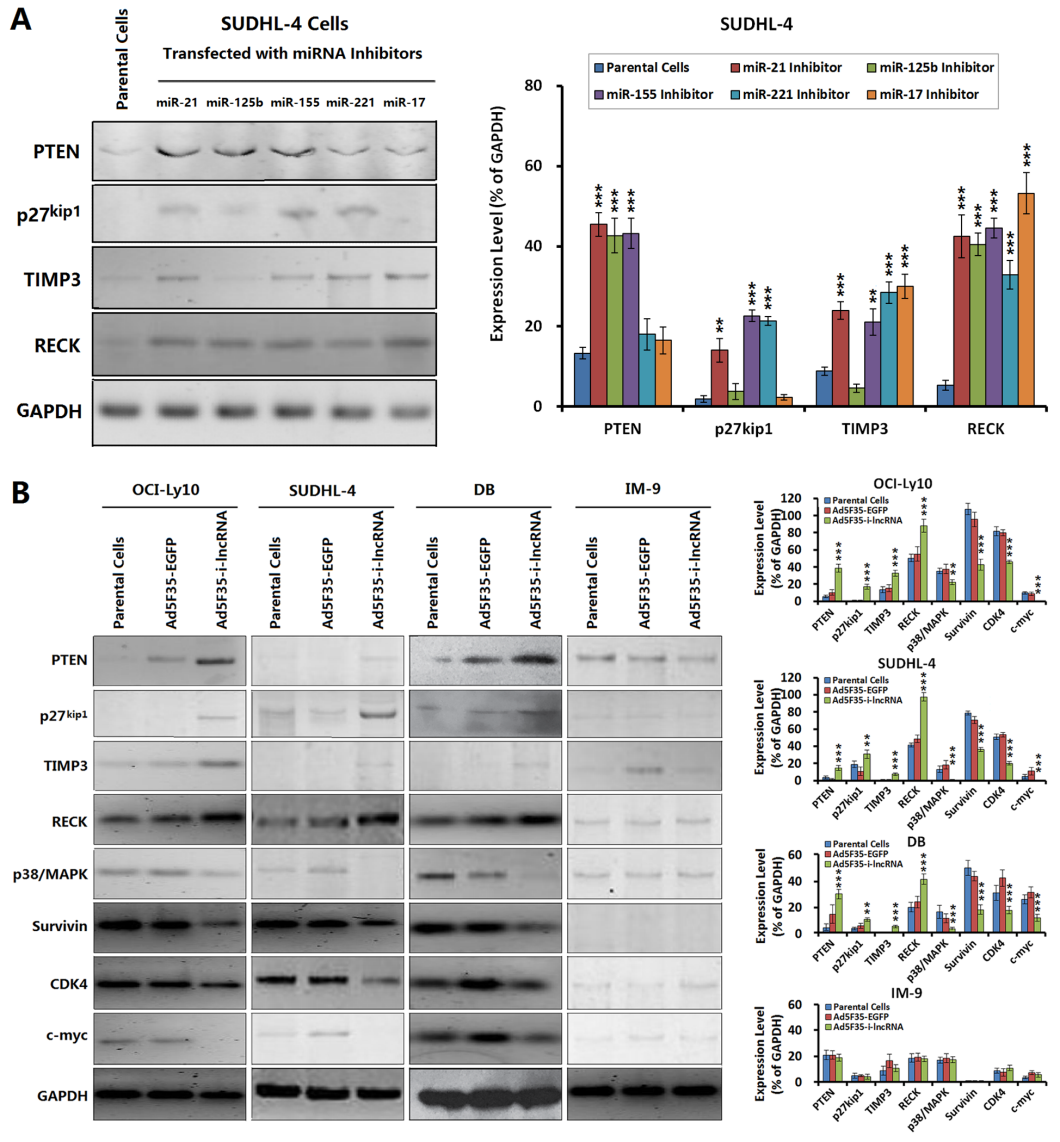
Changes of OncomiR target gene expression mediated by i-lncRNA **A.** SUDHL-4 cells were seeded into 24-well plates at a density of 1×10^6^ cells/100 μL/well and incubated with the indicated miRNA inhibitors at a concentration of 100 nmol/L for 48 h, the harvested cells were managed to detect the expressions of OncomiR target genes. GAPDH was used as the loading control. The densitometry analysis of every protein was performed, normalized with GAPDH content; **P<0.01 and ***P<0.001 compared with the corresponding parental cells. **B.** The four cell lines were planted in 24-well plates at a density of 1×10 ^6^ cells/100 μL/well for 24 h, then infected with Ad5F35-i-lncRNA or Ad5F35-EGFP at an MOI of 100 pfu/cell. After continuously cultured for 48 h, cells were harvested for isolation of total proteins, and the expressions of the indicated proteins were examined by Western blot. The densitometry analysis of every protein was performed, normalized with GAPDH content; **P<0.01 and ***P<0.001 compared with the corresponding parental cells.

When the DLBCL cells were treated with OncomiR inhibitors, the expression levels of some target genes were changed, PTEN was upregulated by the inhibitors for miR-21, miR-125b and miR-155; p27kip1 was upregulated by the inhibitors for miR-21, miR-155 and miR-221; TIMP3 was upregulated by the inhibitors for miR-21, miR-155, miR-221 and miR-17; RECK was upregulated by all the tested inhibitors (Figure 4A). The results demonstrated that every presentative target gene is regulated by more than one miRNA and every miRNA inhibitor can increase the expression of more than one target gene. After confirmed the interactions between OncomiRs and target genes, the DLBCL cells were infected adenovirus Ad5F35-i-lncRNA, consequently, the expression levels of PTEN, p27^kip1^, TIMP3, and RECK were upregulated in DLBCL cells, whereas the expression levels of p38/ MAPK, survivin, CDK4, and c-myc were downregulated (Figure 4B). In contrast, Ad5F35-i-lncRNA-infected IM-9 cells exhibited slight decreases in the expression of p38/MAPK and survivin but no significant changes in the remaining indicators. Ad5F35-EGFP infection induced no significant change in these proteins in any cell line.

### Inhibitory effect of i-lncRNA expression on DLBCL cell xenografts in nude mice

**Figure 5:**
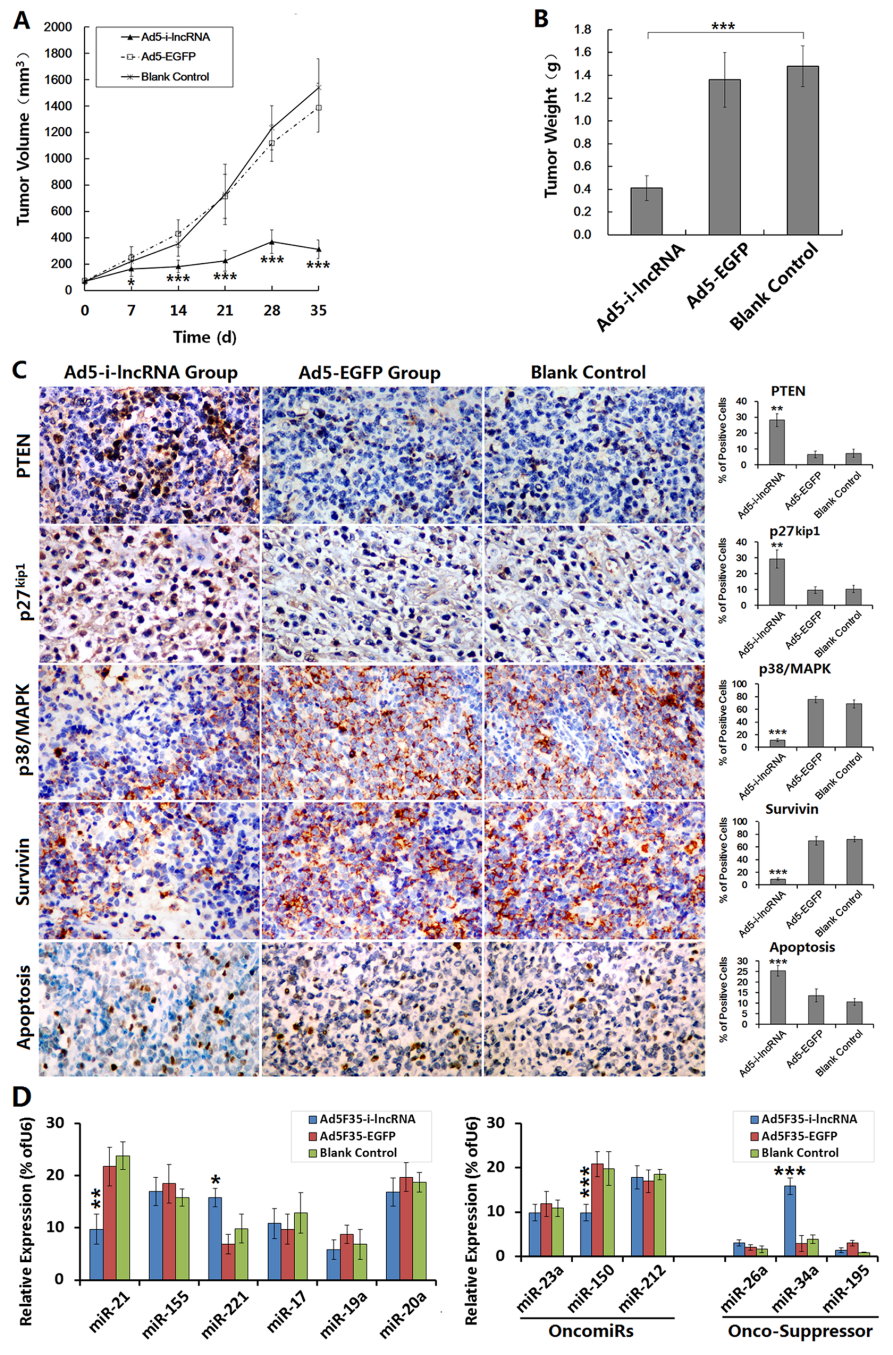
Antitumor efficacy of i-lncRNA on DLBCL xenografts in mouse model **A.** SUDHL-4 cells were used to establish the xenograft tumor models. After tumors had developed, the mice were randomly assigned into three groups (Ad5F35-i-lncRNA group, Ad5F35-EGFP group, blank control group), 10 mice in every group. The virus treatment groups were received 5 injections of total viral dose of 1× 10^9^ pfu/100 μL/mouse, one injection every other day. The blank control group was synchronously received injections of saline with same volume. After treatments, the tumor size was measured weekly and the tumor volume was calculated as “the maximum diameter × minimum diameter^2^ × 0.5” and used to plot growth curves; *P<0.05 and ***P<0.001 compared with the blank control group. **B.** The experimental observation was terminated at day 35 after the first treatment. The tumor specimens were harvested and weighed; ***P<0.001. **C.** The formalin-fixed, paraffin-embedded tumor sections were prepared for immunohistochemical staining to observe the expression of miRNA target gene expression, and the TUNEL assay was performed to evaluate cell apoptosis. For each slice, the positive cells were counted within 5 medium-power magnification fields of view (20× objective lens) under a microscope; original magnification: 200×; **P<0.01 and ***P<0.001 compared with the blank control group. **D.** The fresh tumor tissues were prepared to detect miRNA expression by qRT-PCR; *P<0.05, **P<0.01 and ***P<0.001 compared with the blank control group.

Ad5F35-i-lncRNA and Ad5F35-EGFP were used to treat SUDHL-4 cell xenografts in nude mice. As soon as day 7 after the initial treatment, the tumor growth speed was significantly lower in the Ad5F35-i-lncRNA treatment group than that in the blank control group, however, the tumor continued to grow. The tumor volumes began to decline by day 28 (Figure 5A). Meanwhile, the tumors in the Ad5F35-EGFP and blank control groups continued to grow; by day 35, the tumor volumes in the blank control group exceeded the upper limit of 1,500 mm^3^ permitted by the Ethics Committee of Animal Studies, and thus, the experiment was terminated.

The tumor specimens were removed and weighed; tumors from the Ad5F35-i-lncRNA group weight significantly less than those from the Ad5F35-EGFP and blank control groups (Figure 5B). The tumor specimens were subjected to immunohistochemical staining to analyze the expression of miRNA target gene products, and the results revealed significant increases in the expression levels of PTEN and p27^kip1^ in the Ad5F35-i-lncRNA group and significant decreases in the expression levels of p38/MAPK and survivin. TUNEL labeling indicated a significantly higher apoptosis rate in the Ad5F35-i-lncRNA group (Figure 5C). Although the expressed i-lncRNA competed with the target gene mRNAs to bind to OncomiRs and did not direct change OncomiRs expression, we still detected miRNA expressed in DLBCL xenograft tumors. The i-lncRNA-involved OncomiRs were not always decreased, miR-21 was decreased, miR-221 was increased, and the expression of miR-155, miR-17, miR-19a and miR-20a was not changed, compared with the control group. Interestingly, we also found miR-150 was decreased and miR-34a was increased, suggesting that the expression changes in DLBCL cells after i-lncRNA expression are more complicated (Figure 5D).

## DISCUSSION

DLBCL is the most common type of NHL. Studies have shown that many miRNAs regulate the expression and function of target genes and are thus involved in the development and progression of DLBCL. OncomiRs are a type of miRNA that, upon overexpression, can inactivate certain target genes with tumor suppressor activity and promote the proliferation and metastasis of cancer cells, including DLBCL cells. Studies of clinical DLBCL specimens have shown that upregulated miR-21 expression inhibits the expression of forkhead box protein O1 (FOXO1) and PTEN, thereby increasing the activity of the phosphatidylinositol 3-kinase (PI3K)/protein kinase B (AKT)/mammalian target of rapamycin (mTOR) signaling pathway and imparting more malignant biological characteristics to DLBCL cells. miR-21 overexpression significantly shortens the PFS and OS of DLBCL patients [22]. Many types of malignant tumors, including DLBCL, exhibit high levels of miR-155 expression. In addition, *in vivo* miR-155 transfection has shown to induce lymphoma in murine B cells [23]. miR-155 inactivates the tumor suppressor gene phosphatidylinositol-3,4,5-trisphosphate 5-phosphatase 1 (SHIP1), thereby promoting the tumor necrosis factor (TNF)-α-dependent growth of DLBCL cells [23, 24]. In animal models of DLBCL, miR-155 has been shown to block the transforming growth factor (TGF)-β1-induced activation of retinoblastoma protein (RB), thereby promoting dissociation of the phosphorylated RB (pRB)-E2F1 complex and enabling E2F1 to promote gene transcription and cell cycle progression [25]. High levels of miR-155 also inactivate human germinal center-associated lymphoma (HGAL) and mothers against decapentaplegic homolog 5 (SMAD5) [26, 27], thus increasing the invasiveness of DLBCL cells and leading to a poor clinical prognosis. Strong miR-125a/miR-125b expression directly inhibits the activity of tumor necrosis factor alpha-induced protein 3 (TNFAIP3), thereby activating the NF-κB signaling pathway and promoting the progression of DLBCL [28]. Overexpression of miR-17~92 cluster (miR-17, miR-18a, miR-19a, miR-20a, miR-19b-1, and miR-92-1) induces lymphoma [29]. In addition, malignant B cell proliferation in miR-17~92-overexpressing mice is associated with PTEN and BCL-2-related ovarian death gene (BIM) inhibition [30, 31], as well as PH domain and leucine rich repeat protein phosphatase 2 (PHLPP2) suppression and PI3K/AKT signaling pathway activation [32, 33]. In contrast to OncomiRs, DLBCL cells might also exhibit decreases in or losses of expression of certain miRNAs for which the target genes are oncogenic; accordingly, the decreased expression of these tumor suppressor miRNAs is associated with increased oncogene activity and consequent cancer proliferation, invasion, and metastasis.

In DLBCL cells, changes in miRNA expression levels affect the expression and functions of many target genes and the activity of many signaling pathways, and are thus involved in the development and progression of DLBCL. Accordingly, miRNAs have become promising molecular targets for the treatment of DLBCL. Generally, two miRNA-targeting treatment regimens are available: the use of a miRNA antagonist or inhibitor to suppress OncomiR expression or activity and a targeted increase in the expression of tumor suppressor miRNA [34, 35]. A previous study demonstrated that the *in vivo* administration of a polylysine-conjugated peptide and nucleic acid nanoparticle-coated antisense nucleic acid of miR-155 induced apoptosis and significantly reduced tumor growth in a murine pre-B-cell lymphoma model [36]. Systemic miR-34a administration downregulated FOXP1 expression and induced apoptosis in a DLBCL xenograft mouse model, leading to significant tumor suppressing effect [37]. However, these regimens only target single miRNAs, and thus, their effects are transient and limited. DLBCL is associated with the abnormal expression of multiple genes, as well as different clinical characteristics, treatment responses, and prognoses; this disease involves extensive and complex miRNA regulation processes, allowing cancer cells to easily regain proliferative activity through alternate bypass pathways. Therefore, an intervention strategy simultaneously targeting multiple miRNAs would yield more extensive inhibitory effects and finally provide better outcomes for DLBCL treatment.

Based on our literature review, we selected several OncomiRs proven to be expressed very strongly in DLBCL and generated a tandem sequence containing 10 copies of the complementary binding sequences of these miRNAs; we then used this tandem sequence to design an i-lncRNA and ensured a high copy expression of this molecule in DLBCL cells through adenoviral vector infection and mediation. The i-lncRNA molecules out-competed OncomiRs for binding to target gene mRNAs, thereby consuming large amounts of OncomiRs; this protected the target genes of OncomiRs and enabled many tumor suppressor factors to play an effective anticancer role. The *in vitro* cytology experiments confirmed that the i-lncRNA expression significantly inhibited cell proliferation and induced apoptosis in DLBCL cell lines OCI-Ly10, SUDHL-4, and DB but failed to induce significant effects in normal B lymphocytes. The i-lncRNA expression had different effects on cell cycle phases in different DLBCL cell lines, in OCI-Ly10 cells, the frequencies of cells in the G0/G1 and G2/M phases were increased, whereas the frequency in the S phase was significantly decreased; in contrast, in SUDHL-4 and DB cells, the frequencies of cells were slightly decreased in the G0/G1 phase, increased in the S phase, and significantly decreased in the G2/M phase. An analysis of the OncomiR target gene product expression found significant changes in the protein expression of DLBCL cells with acquired i-lncRNA expression; notably, the expression levels of tumor suppressor genes (PTEN, p27^kip1^, TIMP3, RECK) were upregulated, whereas those of oncogenes (p38/MAPK, survivin, CDK4, c-myc) were downregulated, suggesting that high-copy i-lncRNA expression protected the target genes of OncomiRs. Furthermore, we established SUDHL-4 cell xenografts in nude mice and confirmed the effectiveness of our anticancer strategy in which i-lncRNA was used to competitively consume OncomiRs, thus demonstrating that this treatment strategy involving adenovirus-mediated i-lncRNA expression significantly inhibited the growth of DLBCL xenografts.

Up till now, there is no report about simultaneous intervention of multiple OncomiRs to treat cancer. We have developed a new anticancer intervention strategy that simultaneously targets a group of OncomiRs in DLBCL and have demonstrated that this strategy eliminates the limitations of single miRNA-targeting treatments, thereby significantly improving and prolonging the anticancer effects while exhibiting an excellent safety profile. Our strategy was simultaneously based on multiple highly expressed OncomiRs in DLBCL and could be used as an effective and reliable technology for DLBCL therapy. This group of chose OncomiRs may not be the optimal combination, and further study is necessary to optimize the most effective composition of OncomiRs for DLBCL and other cancers.

## MATERIALS AND METHODS

### Construction of the experimental vectors

A tandem sequence containing 10 copies of complementary sequences to the seed sequences of highly expressed OncomiRs (miR-21, miR-155, miR-221/222, miR-125a-5p/125b, miR-146a/146b-5p, miR-17, miR-19a/19b, miR-20a/20b) in DLBCL (Table 1) was generated and used as the encoding sequence for i-lncRNA. A CACC-box and *Eco*RI site were introduced at the 5′-end, a *Sal*I site was introduced at the 3′-end, and a stop codon (TAA) was introduced into upstream and downstream of the i-lncRNA sequence. The sequence was inserted into the *Eco*RI + *Sal*I sites of plasmid pDC315 (Microbix Biosystems Inc., Mississauga, Ontario, Canada) to construct the i-lncRNA-expressing plasmid pDC315-i-lncRNA. This plasmid and the adenovirus packaging plasmid pAd5F35 were co-transfected into HEK293 cells to recombine the adenovirus Ad5F35-i-lncRNA. Ad5F35-EGFP armed with the enhanced green fluorescent protein (EGFP) gene was used as a negative control adenovirus [38, 39]. The adenoviruses were amplified in large quantities in HEK293 cells and purified via cesium chloride gradient centrifugation. The virus titer was determined through a TCID50 assay. The binding sequence for miR-21 and miR-155 were inserted into the 3′-untranslated region (UTR) of the luciferase vector pGL3-Control (Promega Corporation, Madison, WI, USA) to generate the plasmid pGL3-miRs for the luciferase assay.

**Table 1:** OncomiRs and their complementary binding sequence within i-lncRNA

OncomiRNA	OncomiRNA target genes	OncomiRNA sequence	Complementary binding sequence within i-lncRNA
miR-21	PTEN, RECK, TIMP3, PDCD4, TPM1 [12]	3′ aguuguagucagacuauucgau 5′	5′ ATCAGTCTGATAAGCTA 3′
miR-125a-5p/125b	CDKN2A(p16), TNFAIP3, erbB2/erbB3, VEGF-A, p53, Bak1 [40]	3′ aguguccaauuucccagagucccu 5′ 3′ aguguucaaucccagagucccu 5′	5′ AGTTAAAGGGTCTCAGGGA 3′
miR-155	PTEN, PDCD4, SHIP1 [13] DET1, NIAM, TRIM32, JARID2 [41]	3′ uggggauagugcuaaucguaauu 5′	5′ TATCACGATTAGCATTAA 3′
miR-221/222	p27Kip1(CDKN1B), PTEN, p57Kip2 [14] TIMP3 [42]	3′ cuuugggucgucuguuacaucga 5′ 3′ ugggucaucggucuacaucga 5′	5′ CAGCAGACAGATGTAGCT 3′
miR-17	PTEN, Bim, PHLPP2 [43] TIMP3 [44]	3′ gauggacgugacauucgugaaac 5′	5′ TGCACTGTAAGCACTTTG 3′
miR-20a/20b	PTEN, NCOA3, CAPRIN2 [45] DAPK3, E2F1 [46]	3′ gaugga-cgugauauucgugaaau 5′ 3′ gaugga-cgugauacucgugaaac 5′	5′ GTTTTGCATAGATTTGCACA 3′
miR-146a/146b-5p	ROCK1, TRAF6, IRAK1, CXCR4[47]	3′ uuggguaccuuaagucaagagu 5′ 3′ ucggauaccuuaagucaagagu 5′	5′ CATGGAATTCAGTTCTCA 3′
miR-19a/19b	PTEN, Cyld, Itch, Rnf11, Tax1BP1 [15]	3′ agucaaaacguaucuaaacgugu 5′ 3′ agucaaaacguaccuaaacgugu 5′	5′ CCTGCACTATAAGCACTTTA 3′
Whole-length sequence encoding i-lncRNA	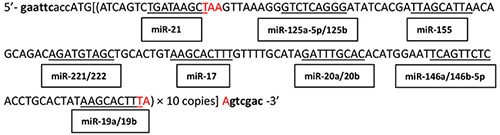		

### Cell line culture and virus infection

DLBCL cell lines (OCI-Ly10, SUDHL-4, DB) were kindly provided by Professor Junmin Li (Department of Hematology, Affiliated Ruijin Hospital of Shanghai Jiao-Tong University, Shanghai, China). A normal human peripheral B cell line (IM-9) was obtained from the Cell Bank of the Institute of Biochemistry & Cell Biology, Chinese Academy of Sciences (Shanghai, China), and cultured following the supplier's instructions. The cell lines were infected with the experimental adenovirus Ad5F35-i-lncRNA or the control adenovirus Ad5F35-EGFP at multiplicities of infection (MOIs) of 10 to 200 plaque-forming units (pfu)/cell to establish virus-infected cell sublines. The luciferase assay was performed in the five cell lines after transfected with OncomiR inhibitors (GenePharma Inc., Shanghai, China) according to the manufacturer's protocol. The vector pGL3-Control was the positive control, and the relative luciferase activity of pGL3-miRs was normalized with the data of pGL3-Control.

### Detection of gene expression

Cell lines including the parental cell lines and cell sublines infected with viruses were cultured for 48 h and harvested. A part of cells were seeded into 24-well plates at a density of 1×10^6^ cells/100 μL/well. After dilution in serum-free medium, a part of cells was incubated with miRNA inhibitors at a concentration of 100 nmol/L for 48 h and harvested for detecting the expressions of OncomiR target genes. Another part of cells was infected with Ad5F35-i-lncRNA and Ad5F35-EGFP at an MOI of 100 pfu/cell in a volume of 100 μL, and the cells were harvested 48 h later. Total RNA was extract for detection of i-lncRNA and miRNA expression by real-time quantitative reverse transcription-polymerase chain reaction (qRT-PCR). The specific PCR primers used for i-lncRNA detection were lncR-F (5′-CACCATGATCAGTCTGATAAG-3′) and lncR-R (5′-TCAGCCCTGAGACCCTAACTC-3′). Total protein was extracted for detection of protein expression by Western blot. Antibodies used for Western blot were purchased from Santa Cruz Biotechnology, Inc. (Santa Cruz, CA, USA), except the mouse anti-Survivin antibody was purchased from Abcam Inc. (Cambridge, MA, USA).

### Detection of cell proliferation

A cholecystokinin-8 (CCK-8) assay was performed to analyze the effect of Ad5F35-i-lncRNA infection on DLBCL cell proliferation; Ad5F35-EGFP was used as a negative viral control, and IM-9 was used as a normal cell control. Cells were harvested in the exponential growth phase, diluted with 10% serum-containing medium, and seeded into 96-well plates at a density of 1 × 10^4^ cells/100 μL/well. After cultured for 24 h, the viruses was diluted with serum-free medium and added to the wells at MOIs of 10 to 200 pfu/cell in a volume of 100 μL/well. Eight replicates were generated for each MOI. The cells were cultured in an incubator for 2 h. Next, the medium was replaced with serum-containing medium (100 μL/well), and the cells were cultured for 48 h followed by CCK-8 assay analysis according to the kit instructions. A microplate reader was used to measure the optical density (OD) of each well at 490 nm, the data were used to plot cell viability curves. The experiments were repeated at an MOI of 100 pfu/cell and at cell culture time for 24 h, 48 h, and 72h.

### Flow cytometric analysis of cell cycle and apoptosis

Cells were infected with Ad5F35-i-lncRNA or Ad5F35-EGFP at a MOI of 100 pfu/cell and harvested 48 h later. A part of cells were centrifuged at 1,000 rpm for 5 min to wipe off cell debris, and fixed in pre-chilled 75% ethanol and placed in a 4°C refrigerator overnight. After two times of wash in phosphate buffered saline (PBS), the RNase-containing propidium iodide (PI) staining mixture was added into the cell suspension, following incubation in dark for 30 min, the cells were subjected to cell cycle analysis by flow cytometry. Another part of cells were stained with Annexin V/PI (Alpha Diagnositic International, San Antonio, TX) and subjected to flow cytometric apoptosis analysis.

### Anti-DLBCL xenograft in mouse model

Forty healthy inbred BALB/C nude mice (20 males and 20 females), 5-week-old, were provided by the Shanghai SLAC Laboratory Animal Center, Chinese Academy of Sciences (Shanghai, China). SUDHL-4 cells were harvested in the exponential growth phase, suspended, and subcutaneously injected into the right armpits of mice at a dose of 1 × 10^7^ cells/100 μL/mouse. Twenty-one days later, tumors had developed in 34 mice (85%) with approximate diameters of 0.5–0.8 cm. The four mice with the two largest and smallest tumors were excluded, and the remaining 30 mice were randomly assigned into three groups (Ad5F35-i-lncRNA group, Ad5F35-EGFP group, blank control group) for a total of 10 mice per group. For the virus treatment groups, the respective adenovirus virus was injected into the tumors at multiple sites with a dosage schedule of five doses at 2 × 10^8^ pfu/100 μL/mouse every 2 days. For the blank control group, saline was injected according to the same dosing schedule (100 μL/mouse/dose). After treatment, the tumor size was measured weekly; the tumor volume was calculated as “the maximum diameter × minimum diameter^2^ × 0.5” and used to plot growth curves.

The experimental observation was terminated if the mean tumor volume in any group exceeded the upper limit of 1,500 mm^3^ permitted by the Ethics Committee of Animal Studies. At termination, 3% sodium pentobarbital was intraperitoneally injected to anesthetize and kill the mice, and tumor specimens were harvested and weighed. A part of fresh tumor tissues was prepared to detect miRNA expression by qRT-PCR. Another part of tumors was fixed in 10% neutral buffered formalin, embedded in paraffin, and sectioned for immunohistochemical staining to observe the expression of miRNA target gene expression (e.g., PTEN [phosphatase and tensin homolog deleted in chromosome 10], p27^kip1^, p38/MAPK, survivin). Terminal deoxynucleotidyl transferase dUTP nick-end labeling (TUNEL) was performed to evaluate apoptosis. For each slice, the number of positive cells was counted within 5 medium-power magnification fields of view (20× objective lens) under a microscope.

### Statistical analysis

The experimental data were statistically analyzed by one-way analysis of variance (ANOVA) and SNK-q test. The software package PASW Statistics 18 was used. The *P* values less than 0.05 were considered statistically significant.
